# Longitudinal Associations of the Healthy Lifestyle Index Score With Quality of Life in People With Multiple Sclerosis: A Prospective Cohort Study

**DOI:** 10.3389/fneur.2018.00874

**Published:** 2018-11-02

**Authors:** Teng I. Leong, Tracey J. Weiland, George A. Jelinek, Steve Simpson, Chelsea R. Brown, Sandra L. Neate, Keryn L. Taylor, Emily O'Kearney, Elasma Milanzi, Alysha M. De Livera

**Affiliations:** ^1^Neuroepidemiology Unit, Melbourne School of Population and Global Health, The University of Melbourne, Melbourne, VIC, Australia; ^2^Menzies Institute for Medical Research, University of Tasmania, Hobart, TAS, Australia; ^3^Biostatistics Unit, Melbourne School of Population and Global Health, The University of Melbourne, Melbourne, VIC, Australia

**Keywords:** multiple sclerosis, lifestyle, risk factors, quality of life, QOL, health outcomes

## Abstract

**Objective:** To explore the association between combined lifestyle risk factors with quality of life in people with multiple sclerosis (MS) over 2.5 years.

**Methods:** People with MS were recruited to participate in a comprehensive online survey regarding their demographic and clinical characteristics, health-related quality of life (HRQOL), and lifestyle behaviors including physical activity, alcohol consumption, cigarette smoking, body mass index, and dietary habits measured at baseline and 2.5-year follow-up. A combined healthy lifestyle index score (HLIS) was constructed by assigning scores of 0–4 to each of the lifestyle risk factors, for which higher values indicate healthier lifestyle behavior. Multivariable linear regression modeling was used to assess whether the HLIS at baseline was associated with the physical and mental HRQOL over the study period in this sample of people with MS.

**Results:** Of 2,466 participants with confirmed MS, 1,401 (57%) completed the follow-up. Multivariable linear regression analyses demonstrated that every 5-point increase (of a possible total of 20) in the baseline HLIS was associated with 1.7 (95% CI: 0.2–3.2) and 2.5 (95% CI: 1.0–4.0) higher scores in the change in physical and mental HRQOL components from baseline to follow-up respectively.

**Conclusion:** Findings suggest the importance of healthy lifestyle behavior in quality of life in MS. A healthy lifestyle program focusing on these behaviors has the potential to positively influence health-related quality of life for people with MS.

## Introduction

Multiple sclerosis (MS) is a chronic disease of the central nervous system (CNS) that affects more than 23,000 people in Australia (1). Characterized by areas of axonal injury and demyelination in the CNS, MS can cause symptoms such as pain, fatigue, muscle weaknesses, impairments to balance, vision, and cognition. All of these can significantly interfere with daily activities and affect health-related quality of life (HRQOL) (2). HRQOL is a multidimensional measure that assesses physical, mental and social functioning and how these domains are affected by disease.

The risk of MS is influenced by a complex interplay of genetic and environmental risk factors (3–8). While susceptibility to MS is influenced by genetics, environmental factors play an important role in the risk for development and progression of the disease. These environmental risk factors comprise of a number of lifestyle risk factors, some of which can be potentially modified.

Several studies have established associations between individual lifestyle risk factors such as physical activity (9), dietary habits (10), BMI (11), alcohol consumption, and smoking (12, 13) with HRQOL in MS. These findings were mostly limited by the cross-sectional study design, which could not reveal the dynamics of association (that is, they could not account for the possibility of reverse causality). Few other studies have examined direct associations of lifestyle risk factors with the progression of MS in terms of HRQOL. One longitudinal study, which included people with relapsing-remitting MS only, found associations between physical activity and HRQOL (14). Another one-year randomized controlled trial of a low-fat, plant-based diet found a statistically significant association with the mental health composite of HRQOL but no association with physical HRQOL (15). Only a small sample of participants (*n* = 61) with limited ethnic diversity were included in this study.

In this study, we adopted a longitudinal observational design, with data collected at two time points from a large international cohort of people with MS. This enabled a prospective examination of the associations between lifestyle risk factors at baseline and HRQOL at the follow-up. To investigate aggregated lifestyle patterns in people with MS, we first generated a healthy lifestyle index score that consisted of the five modifiable-lifestyle risk factors—physical activity, dietary habits, BMI, alcohol consumption, and smoking—modeled on previous scores used in cancer research (16, 17). We then explored the prospective association of this index score at baseline with physical and mental health HRQOL over the study period in this sample of people with MS.

## Methods

### Participants and recruitment

This research involved the analysis of a large international sample of longitudinal data in people with MS who were followed up over a 2.5-year timeframe after baseline surveys undertaken in 2012. The methodology, participant demographics and inclusion/exclusion criteria have been detailed previously (18). In brief, in a general invitation, participants were recruited from MS societies' websites, MS blogs and social media. Participants with self-reported formally diagnosed MS were encouraged to take part. Respondents under 18 years of age were excluded.

### Data collection

An online survey platform, SurveyMonkey™ was used to conduct the survey. Participants read an information sheet before giving consent for continuing the survey. Contact details were recorded to facilitate follow-up. Multiple email reminders were sent to maximize follow-up and for those participants without a valid email address, contact was attempted through alternative means (e.g., Facebook, phone).

The comprehensive online survey consisted of 100 major items at baseline and 111 major items at 2.5 year follow-up and took approximately 40 min to complete. All data were self-reported and stored in a re-identifiable form. Specific items exploring the combined effect of lifestyle risk factors including dietary habits, BMI, physical activity, alcohol consumption, and smoking on the outcome HRQOL were included. Other variables relevant to the analyses were also included, such as gender, age, education, use of disease modifying drugs (DMD) and Patient-Derived MS Severity Score (P-MSSS). Details of these measures are described below.

#### Age

Participants' ages at 2.5 year follow-up were used and calculated with date of birth and date of survey completed.

#### Education

Participants' education levels were categorized into two categories: (a) No formal education or primary school; and (b) secondary school or above.

#### DMD use

Participants were categorized as either (a) currently using DMD (within a provided list of 24 DMDs) or (b) not currently using DMD.

#### P-MSSS

The P-MSSS is an algorithm derived from adjusting disease duration and mean ranks of the Patient-Determined Disease Steps (PDDS) (19). The PDDS is a validated patient-reported outcome of disability in MS focusing mainly on the walking ability of the person and has been shown to correlate with the Expanded Disability Status Scale (EDSS) (20).

#### Dietary habits

Diet quality at the time of the data collection was assessed using a modified version of the Diet Habits Questionnaire (DHQ) (21). It was modified from the original 24-item dietary assessment tool that assesses the type and quality of fat intake and fiber, fruit, vegetable and omega-3 consumption. One item related to alcohol and three items on sodium intake were removed. The rationale for removing these items was that alcohol was measured separately in the survey and sodium intake was not considered a major influence at the time of the baseline survey. A total DHQ score was calculated from the 20-item questionnaire by giving equal weight to all items. Higher scores indicated healthier dietary behavior. The original 24-item tool has been validated in an Australian cardiac disease population (21).

#### BMI

BMI was assessed according to the World Health Organization criteria and defined as a person's weight in kilograms divided by their squared height in meters (kg/m^2^), and was categorized as defined *a priori* (17).

#### Physical activity

Physical activity was assessed with the International Physical Activity Questionnaire Short Form (IPAQ-SF) (22) which measures the frequency and duration of physical activity participation in the past 7 days. A combined total score can be computed to metabolic equivalent of task (MET) minutes per week which has been validated in MS populations (23). Higher values indicated more active lifestyle behavior.

#### Alcohol consumption

Alcohol consumption was measured by frequency and volume in standard drinks. Participants were advised on the study's definition of standard drinks which was equivalent to 10 g of ethanol. This definition included the following examples: one glass of full strength beer (285 ml); two 285 ml glasses of low strength beer; one 100 ml glass of wine; or 30 ml nip or equivalent of mixed spirits. The frequency of alcohol use was collected on an 11-point scale (“never drink” to “drink daily”) and collapsed to a 5-point scale (non-drinker, rarely, <1/week, 1 day/week to 3 days/week, 4 days/week to daily). The volume of alcohol consumption was collected on an 11-point scale (“not applicable” to “10+ standard drinks per day”). Data were then re-calculated to average daily ethanol consumption in grams.

#### Smoking

Smoking was assessed by smoking status (never, current, previous); frequency of smoking among current smokers (15 or less cigarettes per day, or more than 15); and time since quitting among former smokers (10 or more years, or less than 10).

#### Health-related quality of life

HRQOL was assessed using the Multiple Sclerosis Quality of Life-54 questionnaire (MSQOL-54) that was developed from the 36-Item Short Form Health Survey (SF-36) supplemented with 18-items relevant to MS. The MSQOL-54 produces two composite scores for physical health (PHC) and mental health (MHC) determined from a weighted sum of selected subscale scores (24). PHC consists of eight sub-domains namely, physical function, health perceptions, fatigue/energy, physical role limitations, pain, sexual function, social function, and health distress. MHC consists of five sub-domains, namely, cognitive function, emotional role limitations, emotional well-being, health distress, and overall quality of life. Higher scores indicate better quality of life. The questionnaire has been extensively validated with high reliability and strong construct validity across different populations of people with MS (25–27).

### Index construction

In order to quantify the combined impact of lifestyle risk factors, (a) dietary habits, (b) physical activity, (c) alcohol consumption, (d) smoking, and (e) BMI were collected from the data to create a healthy lifestyle index score (HLIS). “Healthier” and “less healthy” behaviors for each lifestyle risk factor in the HLIS were defined *a priori* (17). The cut-offs used along with the modifications are described in detail in the proceeding paragraphs.

The HLIS was constructed from the sum of five lifestyle risk factor component scores by assigning a 5-category score (0 to 4) to each component. The overall health index ranged from 0 to 20, with higher values indicating adherence to a greater number of healthy lifestyle behaviors.

The score for diet was generated by categorizing the values of total DHQ score for the participants into five quintiles. This means that the highest 20% of answers were assigned with the highest score, four, and the lowest 20% answers with the lowest score, zero. The lowest score was defined as having less than 68 in total DHQ score. A score of one was generated for those with 68 to less than 77; a score of two for those with 77 to less than 84; a score of three for those with 84 to less than 91; and a score of four for those with 91 to 100 in total DHQ score.

For BMI, the lowest score, zero, was generated for those with BMI greater than 29, a score of one for BMI between 26 to less than 29; a score of two for BMI between 24 to less than 26, a score of three for BMI between 22 to less than 24; and a score of four for BMI less than 22.

For physical activity, the total MET minutes per week were converted to MET hours per week. The lowest score for physical activity was defined as undertaking less than 45 MET hours per week. Other physical activity quintile scores were defined as (1): between 45 to less than 69 MET hours per week; (2): between 69 to less than 96 MET hours per week; (3): between 96 to less than 134 MET hours per week; and (4): more than 134 MET hours per week.

The healthiest behavior for smoking was defined as “never smoked,” which was assigned a HLIS score of 4. Other categories were defined as (0): current smokers who smoked more than 15 cigarettes per day; (1): current smokers who smoked less than or equal to 15 cigarettes per day; (2): ex-smokers who quit less than 10 years ago; and (3): ex-smokers who quit 10 years or more ago.

For alcohol consumption, a score of 0 was assigned to those consuming an average of greater than or equal to 20 (g/day) of ethanol; a score of 1 was assigned to consuming greater than or equal to 10.0- and less than 20 (g/day); a score of 2 was assigned to consuming an average of greater than or equal to 5 and less than 10 (g/day); a score of 3 was assigned to consuming an average of more than 0 and less than 5 (g/day); and a score of 4 was assigned to those reporting no alcohol consumption.

### Ethics

The original research received ethics approval from the University of Melbourne (HEAG ID: 1545102) and St Vincent's Hospital Melbourne Human Research Ethics Committee (LRR055/12).

### Data analysis

Data were analyzed using Stata, version 13 (StataCorp, College Station, TX, USA). We provided descriptive characteristics of included variables. Continuous variables were summarized using mean and standard deviation and skewed data were presented using median and interquartile range. Categorical variables were summarized using frequency and percentages. For each mental and physical HRQOL component, using linear regression models, we presented an overall prospective analysis. We looked at prospective associations between baseline HLIS and 2.5 year HRQOL with and without adjustment for covariates, but adjusting for baseline HRQOL, and therefore exploring whether the baseline HLIS is associated with the change in HRQOL from baseline to 2.5 years. We identified our covariates *a priori*, using expert subject-matter knowledge based on previous literature (9–12, 28). Using likelihood ratio tests, the assumptions of linear associations between the continuous measures and the outcomes were tested by comparing regression models with categorical (quartile groupings) and pseudo-continuous variables. Analyses were restricted to those with self-reported doctor-diagnosed MS at baseline and complete-case analysis was used (i.e., restricted to participants with complete data). The impact of complete drop-out at follow-up was investigated using summary statistics based on the baseline characteristics of those missing at follow-up.

### Additional analyses of a modified and a rescaled index

Although the exact potential protective mechanism of alcohol use is unclear, several other studies have observed this U or J-shaped association with disability and HRQOL in people with MS (12, 29), as well as with cardiovascular health and total mortality (30, 31). Moderate use of alcohol has also been observed to be associated with a reduction in systemic inflammation (32). As MS has been widely seen as an autoimmune inflammatory disease (33), moderate alcohol usage may potentially positively impact on immune function. As there is no clear evidence for or against moderate alcohol intake in people with MS, two additional analyses were undertaken to investigate the associations of (i) a modified and (ii) a rescaled version of the original HLIS with HRQOL in this cohort of people with MS. In the modified HLIS, a moderate consumption of alcohol (5.0 to < 10 g/day) was assigned with the highest component score of four, while those who never consumed alcohol were assigned with a score of two and highest alcohol consumption (>20 g/day) was assigned with a score of zero. In the rescaled HLIS, the alcohol consumption in the original HLIS was completely removed and the index was rescaled.

## Results

Of 2,466 respondents with confirmed MS, 1401 (56.9%) completed the survey at follow-up. Comparisons between the baseline and follow-up data are shown in Table 1. Of the 1,401 completers, 995 (71.0%) responded to items regarding lifestyle risk factors, allowing the generation of HLIS. The mean HLIS for these participants was 10.2 of a possible total of 20 (SD = 3.4) (Table 1). The distribution of HLIS score is presented in a histogram for respondents at baseline and at 2.5 years (Supplementary Figure 1). With regards to HRQOL, 1,358 (96.9%), and 1,316 (94.0%) completed responses for PHC and MHC respectively. The mean PHC and MHC scores were 60.9 (SD = 34.3) and 70.3 (SD = 20.4) respectively (Table 1).

**Table 1:** Summary statistics of relevant variables.

			**Longitudinal data**			
**Variable**	**Category or numerical range**	**Definition**	**Baseline (n = 2466)**		**2.5 years (n = 1401)**	
			**number**	**Percentage (%) or mean (SD)**	**number**	**Percentage (%) or mean (SD)**
Diet	0	1st quintile (<68)	445	18.05	252	17.99
	1	2nd quintile (68 to < 77)	486	19.71	266	18.99
	2	3rd quintile (77 to < 84)	470	19.06	246	17.56
	3	4th quintile (84 to < 91)	439	17.80	286	20.41
	4	5th quintile (91 to 100)	453	18.37	282	20.13
	Missing	Missing value	173	7.02	69	4.93
BMI	0	5th quintile (>29)	554	22.47	297	21.20
	1	4th quintile (26 to < 29)	314	12.73	176	12.56
	2	3rd quintile (24 to < 26)	345	13.99	204	14.56
	3	2nd quintile (22 to < 24)	448	18.17	270	19.27
	4	1st quintile (<22)	772	31.31	449	32.05
	Missing	Missing value	33	1.34	5	0.36
Physical activity	0	1st quintile (<45 METs/wk)	1518	61.56	937	66.88
	1	2nd quintile (45 to < 69)	228	9.25	136	9.71
	2	3rd quintile (69 to < 96)	134	5.43	78	5.57
	3	4th quintile (96 to < 134)	85	3.45	38	2.71
	4	5th quintile (>134)	106	4.30	53	3.78
	Missing	Missing value	395	16.02	159	11.35
Alcohol	0	1st quintile (≥20 g/day)	143	5.80	79	5.64
	1	2nd quintile (≥10 – <20)	229	9.29	126	8.99
	2	3rd quintile (≥5.0 – <10)	882	35.77	102	7.28
	3	4th quintile (>0 – <5)	571	23.15	567	40.47
	4	5th quintile (Never)	313	12.69	197	14.06
	Missing	Missing value	328	13.30	330	23.55
Smoking	0	1st quintile (current >15 cigs per day)	86	3.49	33	2.36
	1	2nd quintile (current ≤ 15)	193	7.83	62	4.43
	2	3rd quintile (ex-smoker quit <10 years)	450	18.25	204	14.56
	3	4th quintile (ex-smoker quit ≥10 years)	449	18.21	317	22.63
	4	5th quintile (never)	1099	44.57	701	50.04
	Missing	Missing value	189	7.66	84	6.00
Education		Secondary school or above	2397	97.20	1371	97.86
		No formal education or primary school only	55	2.23	10	0.71
		Missing value	14	0.57	20	1.43
Gender		Male	415	16.83	241	17.20
		Female	1937	78.55	1150	82.08
		Missing value	114	4.62	10	0.71
Current DMD use		Yes	1145	46.43	589	42.04
		No	1321	53.57	812	57.96
		Missing value	0	0.00	0	0.00
Country Of Birth		Australasia	682	27.66	464	33.12
		Europe	778	31.55	445	31.76
		North America/Caribbean	881	35.73	412	29.41
		Other	117	4.74	62	4.43
		Missing	8	0.32	18	1.28
Employment		Work or studying	1416	57.42	787	56.17
		Stay at home parent/carer	183	7.42	88	6.28
		Unemployed	208	8.43	85	6.07
		Retired	647	26.24	415	29.62
		Unspecified	2	0.08	3	0.21
		Missing	10	0.41	23	1.64
Disease duration since diagnosis	Range = 0.14 to 42.68	Disease duration since diagnosis	2437	8.07 (7.34)	1401	10.24 (7.07)
Age	Range = 17 to 82	Age for longitudinal group	2428	45.69 (10.48)	1401	48.36 (10.50)
MS Severity (P-MSSS)	Range = 0.42 to 9.98	MS severity scale calculated with years since symptom onset and PDDS	2268	5.01 (2.75)	1352	5.01 (2.73)
HLIS	Range = 0 to 20	The overall HLIS for longitudinal group	1913	10.15 (3.35)	995	10.88 (3.23)
PHC	Range = 0 to 100	Physical health composite score for MSQOL-54	2356	59.70 (33.27)	1358	60.91 (34.34)
MHC	Range = 0.75 to 100	Mental health composite score for MSQOL-54	2270	66.68 (21.35)	1316	70.34 (20.38)

The results of the regression analyses on those who completed the follow-up survey are summarized in Table 2 for the physical health and mental health components. HLIS was included as a continuous term rather than a categorical term in our models as no evidence for non-linearity was found in the association between HLIS and the outcome (likelihood ratio test *p*-values >0.2). A total of 1,105 (~79%) and 1,072 (~77%) respondents who had complete information on covariates and HRQOL composite scores were included in the multivariable regression models for PHC and MHC respectively.

**Table 2:** Associations between baseline HLIS with Physical Health Composite and Mental Health Composite.

**Baseline variables**	**Physical health composite**				**Mental health composite**			
	**Model 1^#^**		**Model2^##^**		Model 1^*^		Model2^**^	
	**Mean difference (95% CI)**	***p*-value**	**Mean difference (95% CI)**	***p*-value**	**Mean difference (95% CI)**	***p*-value**	**Mean difference (95% CI)**	***p*-value**
HLIS	1.61 (0.12, 3.10)	0.03	1.68 (0.22, 3.15)	0.02	3.18 (1.69, 4.66)	<0.001	2.54 (1.04, 4.04)	0.001
Age	−0.02 (−0.29, −0.11)	<0.001	−0.22 (−0.31, −0.12)	<0.001	−0.10 (−0.19, −0.02)	0.02	−0.05 (−0.14, 0.04)	0.26
Female gender	0.24 (−2.06, 2.55)	0.84	−0.30 (−2.72,2.12)	0.81	−0.18 (−2.47, 2.11)	0.88	0.37 (−2.05,2.78)	0.77
Secondary or higher education	−2.27 (−11.51,6.96)	0.63	1.92 (−9.66,13.50)	0.75	8.67 (−1.11,18.45)	0.08	2.99 (−8.48,14.46)	0.61
P-MSSS	−1.63 (−2.12, −1.15)	<0.001	−1.48 (−2.00, −0.96)	<0.001	−0.66 (−0.99, −0.33)	<0.001	−0.55 (−0.91, −0.82)	0.003
DMD use	1.27 (−0.50, 3.03)	0.16	2.36 (0.48, 4.24)	0.01	0.56 (−1.18, 2.31)	0.53	0.97 (−0.93, 2.86)	0.32

# *Individual associations adjusted for baseline Physical Health Composite only*.

## *Associations adjusted for the covariates presented in the table and the baseline Physical Health Composite. Complete-case analysis: Physical Health Composite: N = 1,105*.

* *Individual associations adjusted for baseline Mental Health Composite only*.

** *Associations adjusted for the covariates presented in the table and the baseline Mental Health Composite. Complete-case analysis: Mental Health Composite: N = 1,072*.

### Physical health composite

A strong positive association of HLIS with PHC was observed. Every 5-point increase in the HLIS at baseline was associated 1.7 (95% CI: 0.2–3.2) higher scores in the change in physical HRQOL component from baseline to follow-up. Models were adjusted for age, gender, education, P-MSSS, and DMD use.

### Mental health composite

HLIS also had strong positive associations with the mental health composite of MSQOL-54. Every 5-point increase in the baseline HLIS was associated with 2.5 (95% CI: 1.0–4.0) higher scores in the change in mental HRQOL component from baseline to follow-up. Models were adjusted for age, gender, education, P-MSSS, and DMD use.

Additional analyses of the associations between original HLIS and the two HRQOL composite scores are presented in Supplementary Table 1. Compared to the original HLIS, for PHC, similar effect sizes were observed using modified HLIS, however the effect size attenuated when the rescaled HLIS with the complete removal alcohol consumption was used. For MHC, a slightly larger effect size was observed using the modified HLIS, however the effect size stayed similar when the rescaled HLIS was used. For completeness, we presented baseline characteristics of completers and non-completers at follow-up in Supplementary Table 2. A detailed comparison of these characteristics has been described elsewhere (34), where it has been shown that the completion of the 2.5-year wave is associated with healthier lifestyle and better health outcomes. In Supplementary Table 3, we have also presented baseline characteristics according to baseline HLIS quartiles. Compared to people with MS in the first quartile, those in the fourth quartile tend to be younger and have lower disease severity.

## Discussion

### Summary of main findings

Our data, based on a large international cohort, predominantly Western and English-speaking people with MS, revealed strong longitudinal associations between healthier lifestyle and better HRQOL in terms of physical and mental health composite scores of the MSQOL-54. This is the first longitudinal study to explore the effect of aggregated lifestyle risk factors on HRQOL in people with MS. Overall, the study participants appeared to have moderately healthy lifestyle, as indicated by the mean scores on the modified HLIS (Table 1, Supplementary Figure 1).

Our focus on HRQOL may be considered a strength of the study. Due to the incomplete reflection of disease burdens by traditional clinical endpoints such as physical disability, HRQOL has gained increasing clinical importance in MS management (35, 36). Indeed, MS poses significant psychological strains which are often overlooked and undertreated (37). The use of HRQOL provides a more sensitive indication of overall wellbeing and enables better disease management. HRQOL has been designated as an important outcome for treatment of chronic diseases by the World Health Organization (38). Overall, HRQOL is a valuable measure that is a major outcome for intervention programs to target.

Although potentially difficult to change, most lifestyle risk factors can be modified. Modifiable risk factors such as higher body mass index (BMI), serum lipid profile and smoking have been found to be associated with a more rapid rate of disability progression (39, 40). Consolidated recent evidence (41) highlights the important role of modifiable risk factors and presents opportunities for intervention programs to improve disease management and secondary prevention of MS. It has been shown that common lifestyle risk factors cluster among adults in the general population and this tendency for risk factors to aggregate can have important implications for health promotion (42). While for several high-burden diseases such as colorectal cancer and breast cancer, the impact of aggregated lifestyle factors on health outcomes has been explored previously (16, 17), no study to date has investigated this in people with MS. This research attempts to fill a gap in the MS literature where there is a need for longitudinal studies of people with MS where aggregated modifiable lifestyle risk factors can be explored to provide more informative epidemiological findings and translation to preventive interventions in randomized controlled trials.

### Limitations

Nonetheless, our findings had several limitations. First, our complete case analyses were based on the assumption that data were missing completely at random. The comparison of baseline characteristics for those who did not complete to those that completed the follow-up suggested that this may not be a realistic assumption. There seem to be a higher proportion of participants who did not complete the follow-up at relatively lower levels of HLIS (less healthy) (See Supplementary Table 1). However, in this study, we lack auxiliary variables—the variables that are highly correlated with the variables with missing values, but are not in the analysis model of interest. In this setting, we are less likely to gain information about the exposure and outcome association from approaches to handling missing values such as multiple imputation, given that the missing values are in our outcome variables (43). Imputation approaches can result in biased inference if not carried out appropriately or if the underlying assumptions are not justifiable (44). Further, this cohort was recruited through an online methodology which may not reflect health behaviors in the general population of people with MS. For example, differences in health behaviors were identified when comparing our study findings to a survey from the North American Research Committee on Multiple Sclerosis Registry (45). Our study participants from both cohorts appeared to be moderately healthy, yet survey results from the registry suggested otherwise (45). This could imply disproportionate lifestyle risk factors between respondents and the general population of people with MS. As such, non-response or participation bias limits the generalizability of our findings to the global community of people with MS.

In our sample, those that did not complete the follow-up were more likely to have missing responses at baseline (See Supplementary Table 2). This could imply those that were lost to follow-up may have had little interest in health while those completing follow-up may have been more health-conscious. It is possible that the health-conscious respondents were more likely to report healthier lifestyle behaviors. The accuracy of our data may have been affected as all of the responses were self-reported. Although most of the variables were measured with validated tools, the lack of verification regarding diagnosis of participants can still affect, to some extent, the reliability of our data. Some of the variables were also hard to measure. Particularly, when respondents were asked to recall certain information such as dietary habits, the amount of alcohol consumption and duration of physical activity in the past 7 days. In addition, our data may also have been affected by social desirability bias, as the participants may tend to overreport healthier behaviors and underreport detrimental behaviors.

The range of lifestyle behavior measured in the original study was based on existing literature that support the relationships with MS health outcomes. However, due to the limitation of using pre-existing data from the original study, we were unable to include additional or alternative information that could have been relevant to our study. For instance, sodium intake was excluded from the DHQ questionnaire, yet the relationship between consumption of sodium and disease progression in people with MS is unclear. One observational study found positive associations (higher intake in sodium correlated with increased disease activity in people with MS) (46) while another found no associations (47). The exclusion of sodium intake could have affected the estimated associations in both directions. In addition, the questionnaire assigned margarine use with favorable scoring, for example. Margarine is a source of industrially produced trans fatty acids (48) and trans fatty acids have been reported to be associated with up-regulation of pro-inflammatory cytokines (49). As elevation of these cytokines is evident in the involvement of MS disease activity, the favorable scoring for margarine use should be reconsidered when using the DHQ in people with MS (50). Consequently, the lack of robustness of the questionnaire; its lack of validation in MS populations; and its applicability across ethnic diversities limit the strength of our findings on the contribution of diet to health outcomes in people with MS.

Similar issues can be found in the assessment of physical activity with HRQOL. It is possible that certain types or intensities of physical activity have different effects in people with different levels of disability. However, the instrument (IPAQ-SF) used to measure physical activity in people with MS lack the relevant measurement of an individual's ability to participate in physical activity, and it cannot be concluded that which form of physical activity is most beneficial to people with MS.

The construction of HLIS also has its limitations. In the HLIS, different lifestyle risk factors were weighted equally. The choice is an imperfect estimation that each lifestyle behavior has equal impact on HRQOL. In addition, for the purpose of this study, we adhered to the original cut-off points whenever possible. The cut-off thresholds for physical activity were pre-defined from a very large sample (*n* = 242, 918) of menopausal women (17). However, the top quintiles of physical activity may not be achievable by the general MS population. This is because the level of physical activity depends on the level of disability, and people with MS are often affected by symptoms such as pain which prevents participation in physical activity (51). Finally, former smoking behavior is a past event. This implies that the HLIS has limited power to reflect on the combined impact of *modifiable* risk factors, but rather, only on lifestyle risk factors.

### Recommendations and implications

In light of these limitations, several recommendations for future studies can be made. Interviewer-administered 24-h recall in future assessment may be incorporated to measure usual frequency in physical activity, food and fluid intakes. Better validation of the DHQ tool on the MS population or using a more comprehensive tool for the assessment of diet on MS morbidity may also be useful. While having a more comprehensive tool allows better capture on the complexities of lifestyle patterns, it also imposes a burden to survey respondents. In order to reduce drop out or non-response bias, it is important to keep a balance between concise and comprehensive questionnaires. By minimizing these biases, more accurate assessments of lifestyle risk factors may be obtained which can be crucial for advancing the internal validity of the study.

Future studies can also seek to validate the positive associations we have seen in this study on a general population of people with MS. More studies are warranted to investigate the causal relationship between HLIS and HRQOL in people with MS. In particular, randomized controlled trials could be undertaken in order to examine this issue. More studies are also required to understand the underlying biological mechanisms between different lifestyle behaviors, complex interplay between the constructs of HLIS and other variables (52) and HRQOL, thereby allowing the examination of potential influence of different weightings in the construction of HLIS.

Nonetheless, this study highlights the importance of lifestyle behaviors on HRQOL in people with MS. This provides opportunities for future targeted lifestyle interventions to improve the morbidity of people with MS.

## Conclusions

Healthier lifestyle behavior is associated with better mental and physical health related quality of life in this longitudinal sample of people with MS. These findings are highlight the importance of health promotion programs to improve chronic disease management in people with MS, as in other chronic diseases. Further research is needed to establish causal relationships as well as predicting changes in HRQOL throughout the course of MS.

## Author contributions

TL is involved in investigation, data curation, data analysis, and writing the original draft. TW and AD are involved in supervision. All authors are involved in the conceptualization, methodology, review, and editing.

### Conflict of interest statement

GJ receives royalties for his book Overcoming Multiple Sclerosis; GJ, SN, and KT have received remuneration for facilitating residential retreats outlining the research behind a secondary preventive lifestyle modification approach to MS management. The remaining authors declare that the research was conducted in the absence of any commercial or financial relationships that could be construed as a potential conflict of interest.
