# Supplementary material for: Longitudinal Associations of the Healthy Lifestyle Index Score With Quality of Life in People With Multiple Sclerosis: A Prospective Cohort Study
Source: Front Neurol. 2018 Nov 2;9:874. doi: 10.3389/fneur.2018.00874 (PMC6225868; doi:10.3389/fneur.2018.00874)
Supplement: Supplementary file 1 [file Table_1.docx]

**Supplementary Table 1.** Association between baseline (a) original, (b) modified (c) rescaled HLIS with PHC and MHC at 2.5 year follow-up.

| **Outcome variables** | **Adjusted model*** | | | | | |
| --- | --- | --- | --- | --- | --- | --- |
|  | **Original alcohol consumption** | | **Modified alcohol consumption** | | **No alcohol consumption** | |
|  | **Mean difference (95% CI)** | **p-value** | **Mean difference (95% CI)** | **p-value** | **Mean difference (95% CI)** | **p-value** |
| **PHC** | 1.69 (0.22,3.20) | 0.02 | 1.68 (0.22,3.15) | 0.02 | 1.51 (0.23,2.79) | 0.02 |
| **MHC** | 2.54 (1.04 , 4.04) | 0.001 | 2.80 (1.33 ,4.28) | <0.001 | 2.59 (1.31,3.88) | <0.001 |

^*^per 5 increase in the original, modified, or rescaled HLIS. Associations adjusted for the baseline variables and the baseline QOL. Complete-case analysis: PHC: N=1105; MHC: N=1072. Age, P-MSSS, HLIS were continuous measures, while DMD use, gender, education were binary measures.
