# Supplementary material for: Longitudinal Associations of the Healthy Lifestyle Index Score With Quality of Life in People With Multiple Sclerosis: A Prospective Cohort Study
Source: Front Neurol. 2018 Nov 2;9:874. doi: 10.3389/fneur.2018.00874 (PMC6225868; doi:10.3389/fneur.2018.00874)
Supplement: Supplementary file 2 [file Table_2.docx]

**Supplementary Table 2.** Baseline characteristics of participants who completed and dropped out at 2.5 year follow-up.

| **Baseline characteristic** | **Sample of participants that completed the follow-up (N=1401)** | | | **Sample of participants that dropped out at follow-up (N=1065)** | | |
| --- | --- | --- | --- | --- | --- | --- |
|  | **number or mean** | **Percentage or standard deviation** | **Missing percentage at baseline** | **number or mean** | **Percentage or standard deviation** | **Missing percentage at baseline** |
| **Gender, n (%)** |  |  | 0.71% |  |  | 9.77% |
| Male | 241 | 17.20% |  | 174 | 16.37% |  |
| Female | 1150 | 82.08% |  | 787 | 74.04% |  |
| **Age, mean (SD)** | 45.93 | 10.48 | 0% | 45.36 | 10.49 | 3.57% |
| **Education status, n (%)** |  |  | 0.64% |  |  | 0.47% |
| Secondary school or above | 1378 | 98.36% |  | 1019 | 95.68% |  |
| No formal education or primary school only | 14 | 1% |  | 41 | 3.85% |  |
| **Current DMD use** |  | 0% |  |  |  | 0% |
| Yes | 654 | 46.68% |  | 491 | 46.10% |  |
| No | 747 | 53.32% |  | 574 | 53.90% |  |
| **Country of birth** |  |  | 0.21% |  |  | 0.47% |
| Australasia | 466 | 33.26% |  | 216 | 20.28% |  |
| Europe | 448 | 31.98% |  | 330 | 30.99% |  |
| North America/ Caribbean | 421 | 30.05% |  | 460 | 43.19% |  |
| Other | 63 | 4.50% |  | 54 | 5.07% |  |
| **Employment** |  |  | 0.21% |  |  | 0.66% |
| Work or studying | 862 | 61.53% |  | 554 | 52.02% |  |
| Stay at home parent/carer | 101 | 7.21% |  | 82 | 7.70% |  |
| Unemployed | 103 | 7.35% |  | 105 | 9.86% |  |
| Retired | 332 | 23.70% |  | 315 | 29.58% |  |
| Unspecified | 0 | 0% |  | 2 | 0.19% |  |
| **Disease duration since diagnosis** | 7.73 | 7.06 | 0% | 8.53 | 7.69 | 2.72% |
| **P-MSSS** | 4.80 | 2.73 | 4.71% | 5.32 | 2.75 | 12.39% |
| **Diet, n (%)** |  |  | 3.93% |  |  | 11.08% |
| 1st quintile (< 68) | 201 | 14.35% |  | 244 | 22.91% |  |
| 2nd quintile (68 to <77) | 257 | 18.34% |  | 229 | 21.50% |  |
| 3rd quintile (77 to <84) | 269 | 19.20% |  | 201 | 18.87% |  |
| 4th quintile (84 to <91) | 270 | 19.27% |  | 169 | 15.87% |  |
| 5th quintile (91 to 100) | 349 | 24.91% |  | 104 | 9.77% |  |
| **Body mass index, n (%)** |  |  | 0.36% |  |  | 2.63% |
| 5th quintile (>29) | 271 | 19.34% |  | 283 | 26.57% |  |
| 4th quintile (26 to <29) | 165 | 11.78% |  | 149 | 13.99% |  |
| 3rd quintile (24 to <26) | 213 | 15.20% |  | 132 | 12.39% |  |
| 2nd quintile (22 to <24) | 283 | 20.20% |  | 165 | 15.49% |  |
| 1st quintile (<22) | 464 | 33.12% |  | 308 | 28.92% |  |
| **Physical activity, n (%)** |  |  | 11.28% |  |  | 22.25% |
| 1st quintile (<45 METs/wk) | 914 | 65.24% |  | 604 | 56.71% |  |
| 2nd quintile (45 to <69) | 134 | 9.56% |  | 94 | 8.83% |  |
| 3rd quintile (69 to <96) | 81 | 5.78% |  | 53 | 4.98% |  |
| 4th quintile (96 to <134) | 51 | 3.64% |  | 34 | 3.19% |  |
| 5th quintile (>134) | 63 | 4.50% |  | 43 | 4.04% |  |
| **Alcohol, n (%)** |  |  | 9.21% |  |  | 18.69% |
| 1st quintile (≥20 g/day) | 88 | 6.28% |  | 55 | 5.16% |  |
| 2nd quintile (≥10 - <20) | 144 | 10.28% |  | 85 | 7.98% |  |
| 3rd quintile (Never) | 461 | 32.91% |  | 421 | 39.53% |  |
| 4th quintile (≥0 - <5) | 368 | 26.27% |  | 203 | 19.06% |  |
| 5th quintile (≥5.0 - <10) | 211 | 15.06% |  | 102 | 9.58% |  |
| **Smoking, n (%)** |  |  | 4.71% |  |  | 11.55% |
| 1st quintile (current >15 cigs per day) | 32 | 2.28% |  | 54 | 5.07% |  |
| 2nd quintile (current ≤15) | 81 | 5.78% |  | 112 | 10.52% |  |
| 3rd quintile (ex-smoker quit <10 yrs) | 236 | 16.84% |  | 214 | 20.09% |  |
| 4th quintile (ex-smoker quit ≥10 yrs) | 279 | 19.91% |  | 170 | 15.96% |  |
| 5th quintile (never) | 707 | 50.46% |  | 392 | 36.81% |  |
| **Health related QOL (MSQOL-54), mean (SD)** |  |  |  |  |  |  |
| Physical Health Composite | 63.46 | 32.34 | 3.00% | 54.57 | 33.84 | 6.38 |
| Mental Health Composite | 70.29 | 19.57 | 5.28% | 61.60 | 22.67 | 11.46% |
| **HLIS** | 10.70 | 3.31 | 17.06% | 9.30 | 3.22 | 29.48% |
