# Supplementary material for: Longitudinal Associations of the Healthy Lifestyle Index Score With Quality of Life in People With Multiple Sclerosis: A Prospective Cohort Study
Source: Front Neurol. 2018 Nov 2;9:874. doi: 10.3389/fneur.2018.00874 (PMC6225868; doi:10.3389/fneur.2018.00874)
Supplement: Supplementary file 3 [file Table_3.docx]

**Supplementary Table 3: Baseline characteristics according to quartiles of the HLIS**

| **Baseline Characteristic** | **First quartile** | **Second quartile** | **Third Quartile** | **Fourth Quartile** |
| --- | --- | --- | --- | --- |
| Age^#^ | 46.2 (9.8) | 46.4 (10.8) | 45.1 (10.9) | 44.2 (10.4) |
| Female gender^##^ | 419 (83.6) | 509 (80.4) | 339 (83.5) | 309 (84.9) |
| Secondary or higher education^##^ | 485 (97.2) | 624 (98.6) | 406 (98.0) | 358 (98.0) |
| P-MSSS^#^ | 5.6 (2.7) | 5.2 (2.8) | 4.7 (2.7) | 4.1 (2.5) |
| DMD use^##^ | 275 (54.8) | 327 (51.7) | 202 (49.2) | 179 (48.7) |

**^#^**Mean (standard deviation), ^##^Number(Percentage)
