# Supplementary material for: Longitudinal Associations of the Healthy Lifestyle Index Score With Quality of Life in People With Multiple Sclerosis: A Prospective Cohort Study
Source: Front Neurol. 2018 Nov 2;9:874. doi: 10.3389/fneur.2018.00874 (PMC6225868; doi:10.3389/fneur.2018.00874)
Supplement: Supplementary file 4 [file Image_1.pdf]

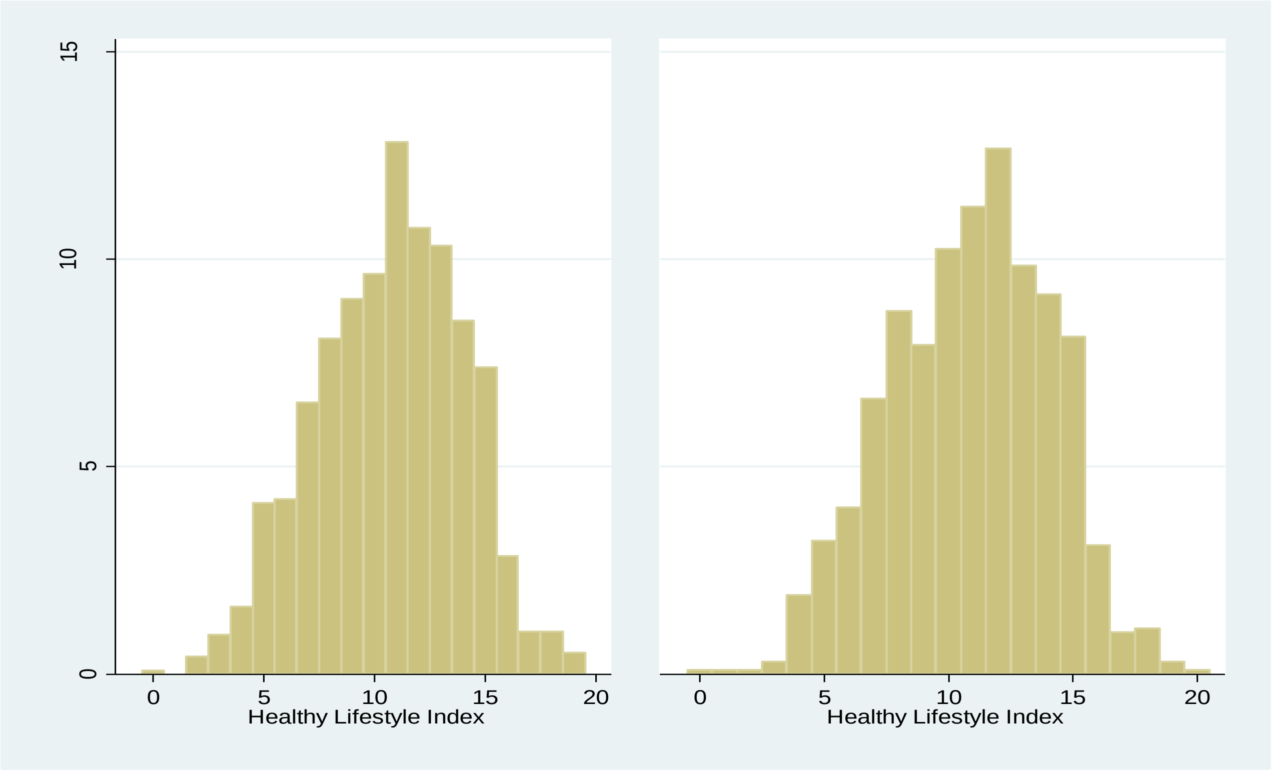

Supplementary Figure 1. Distribution of HLIS for longitudinal cohort (n=1401) at baseline (left) and follow-up (right).
